# A Six-Month Supplementation of Mulberry, Korean Red Ginseng, and Banaba Decreases Biomarkers of Systemic Low-Grade Inflammation in Subjects with Impaired Glucose Tolerance and Type 2 Diabetes

**DOI:** 10.1155/2012/735191

**Published:** 2012-02-28

**Authors:** H.-J. Kim, K.-H. Yoon, M.-J. Kang, H.-W. Yim, K.-S. Lee, V. Vuksan, M.-K. Sung

**Affiliations:** ^1^Department of Food and Nutrition, Sookmyung Women's University, Yongsan-gu, Seoul 140-742, Republic of Korea; ^2^Department of Endocrinology and Metabolism, Seoul St. Mary's Hospital The Catholic University of Korea, Seocho-gu, Seoul 137-701, Republic of Korea; ^3^Department of Preventive Medicine, Seoul St. Mary's Hospital The Catholic University of Korea, Seocho-gu, Seoul 137-701, Republic of Korea; ^4^Korea Ginseng Manufacturing Plant, National Agricultural Cooperative Federation, Chung-buk 368-811, Republic of Korea; ^5^Clinical Nutrition & Risk Factor Modification Center, St. Michael's Hospital and, Department of Nutritional Sciences, University of Toronto, Toronto, ON, Canada M5C 1N8

## Abstract

We sought the long-term efficacy of traditionally used antidiabetic herbs in controlling blood glucose homeostasis and low-grade inflammation. Ninety-four subjects with either impaired glucose tolerance or mild T2D were randomized either to treatment arm or placebo arm and received 1 : 1 : 1 mixture of ginseng roots, mulberry leaf water extract, and banaba leaf water extract (6 g/d) for 24 weeks. Oral 75 g glucose tolerance test was performed to measure glucose and insulin responses. Blood biomarkers of low-grade inflammation were also determined. Results found no significant difference in glucose homeostasis control measure changes. However, plasma intracellular adhesion molecule-1 (ICAM-1) concentration was decreased showing a significant between-treatment changes (*P* = 0.037). The concentrations of vascular cell adhesion molecule-1 (VCAM-1) (*P* = 0.014) and ICAM-1 (*P* = 0.048) were decreased in the treatment group at week 24, and the oxidized low-density lipoprotein (ox-LDL) concentration was reduced at week 24 compared to the baseline value in the treatment group (*P* = 0.003). These results indicate a long-term supplementation of ginseng, mulberry leaf, and banaba leaf suppresses inflammatory responses in T2D.

## 1. Introduction

Type 2 diabetes (T2D) is the fastest growing metabolic disorder in many parts of the world. The incidence of T2D in 2000 is ~171 million worldwide, and this number is estimated to be doubled in 2030 [1]. T2D has higher risk of developing vascular complications which increase the risk of cardiovascular diseases (CVDs). Recent studies have also suggested that impaired glucose tolerance is a risk factor for CVD independent of the subsequent T2D development [2, 3]. Therefore, the reversal of impaired glucose tolerance (IGT) to normal glucose tolerance (NGT) is an important strategy to prevent both T2D and CVD.

The frequent development of type 2 diabetes and CVD among obese population is possibly related to excess adiposity and subsequent low-grade chronic inflammation [4, 5]. The elevated concentrations of circulating free fatty acids and inflammatory mediators in obese subjects create insulin resistance [6, 7] which is associated with a cluster of metabolic abnormalities including dyslipidemia, hyperglycemia, and hypertension [8] followed by the development of premature atherosclerosis as well as type 2 diabetes. The postprandial glucose excursion has been suggested as an independent factor to activate thrombotic conditions through a transient overproduction of reactive oxygen species [9].

A growing number of people with T2D use alternative therapies including herbal medicines, and many of them are not proven for their efficacies. Ginseng has been used for many years as one of the most popular folk herbal remedies to manage symptoms of diabetes. However, it is not until recent years to provide scientific evidence that ginseng possesses blood glucose control activities possibly by enhancing insulin sensitivity [10]. We have previously reported that Korean red ginseng root, mixed with mulberry leaves and banaba leaves, exerts efficient regulation of blood glucose homeostasis in db/db mouse [11]. Mechanistic explanations included the upregulation of liver PPAR-*α*, adipose tissue PPAR-*γ*, and lipoprotein lipase (LPL) gene expression, which improved insulin sensitivity and lipid metabolism. Mulberry leave extract has been shown to possess hypoglycemic effects in an IDDM animal model, and a potential hypoglycemic compound in mulberry leaves was suggested as 2-*O*-*α*-*D*-galactopyranosyl-DNJ) and fagomine [12]. Banaba leaf extract has been shown to regulate blood glucose concentration, and active compounds are identified as corosolic acid and penta-*O*-galloyl-glucopyranose [13] which improve lipid metabolism or glucose uptake, respectively. Therefore the hypothesis of this study was the supplementation of red ginseng roots, mulberry leaf water extract, and banaba leaf water extract for 6 months improves blood glucose homeostasis by increasing insulin sensitivity and suppresses low-grade inflammation in subjects with impaired glucose tolerance or mild cases of T2D.

## 2. Materials and Methods

### 2.1. Study Population

Individuals with impaired glucose tolerance and mild cases of T2D were recruited based on the report of the Expert Committee on the Diagnosis and Classification of Diabetes Mellitus [14] Eligibility criteria included 35–79 years old, BMI over 20 kg/m^2^, and nonpregnant who has not taken herbs at least for the last 3 months. Exclusion criteria were patients taking insulin or medications other than sulfonylurea, biguanide, or *α*-glucosidase during the last 2 months individuals with chronic liver diseases, advanced kidney diseases, atherosclerosis, myocardial infarction, pulmonary diseases, gastrointestinal diseases, hematological diseases, and cancer. Individuals receiving or have received pharmacological doses of steroids and/or participating in weight reduction programs were also excluded. The sample size was calculated based on a previous clinical trial [10], and 80 min·mmol/L reduction in the total area under the curve of glucose during the oral glucose tolerance test (OGTT AUCg) was considered to be the primary efficacy point with the average standard deviation (SD) of 105 min·mmol/L at a two-tailed alpha = 0.05 and 1-*β* = 90%. The study was approved by the Seoul St. Mary's Hospital ethics review board, and eligible participants gave informed written consent.

### 2.2. Study Design

**Figure 1:**
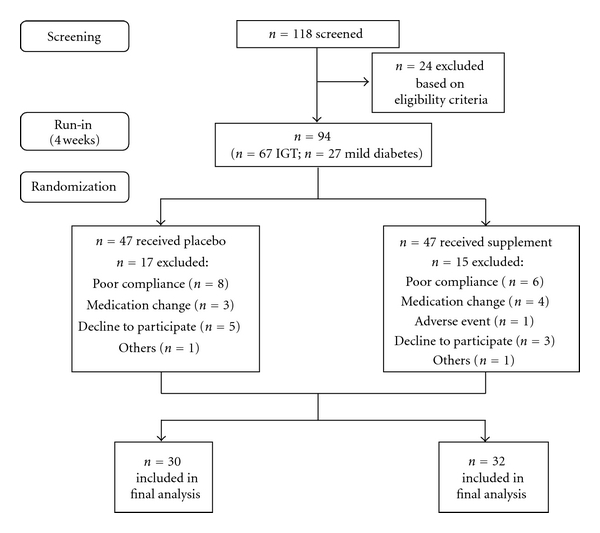
Schematic of the flow of study participants. A total of 118 individuals agreed to participate were screened, and 94 subjects began on the placebo run-in period for 4 weeks to be acclimatized to the treatment protocol and stabilize baseline measures. The study subjects were randomly allocated to either placebo group or supplement group. Final analyses included 30 subjects in placebo group and 32 subjects in supplement group.

The study used a randomized, placebo-controlled, double-blind, parallel design. The enrolled subjects began on the placebo run-in period for 4 weeks to be acclimatized to the treatment protocol and stabilize baseline measures (Figure 1). The treatment protocol was determined from the previous Korean red ginseng clinical trial [10]. The total treatment dose was 6 g/day (2 g × 3 times taken 40 min before each meal). Placebo capsules were prepared with cornstarch. Treatments were concomitant with usual therapy, and dietary counseling was provided based on the guideline of the Korean Society of Diabetes/Korean Association of Dietetics. The treatment period was 6 months, and each enrolled participant visited the hospital every 3 months to have clinical and biochemical measurement taken, receive next treatment capsules, and return unused capsules. Three-day dietary records were collected during the last three days before visit 1 (baseline) and visit 3 (week 24). Demographics measures, anthropometric measures, and exercise habits were obtained from study participants at all visits. The type and dose of oral medication were maintained throughout the study. Compliance measures included the number of returned capsules, body weight, and 3-day dietary records which was reviewed by a trained dietitian using food and portion size models.

### 2.3. Preparation of Supplements

The Korean red ginseng (Panax ginseng C.A. Mayer) powder, mulberry (*Morus alba* L.) leaf water extract powder, and banaba (*Lagerstroemia* speciosa L.) leaf water extract powder were kind gifts from the National Agricultural Cooperative Federation (Jeung-pyeong, Republic of Korea). Three herbal preparations were mixed in equivalent ratio and encapsulated (500 mg/capsule). The preparation procedures for each herb are described in the previous report [11]. Quality control of the herb mixture was based on the contents of the marker substances which are total ginsenosides for red ginseng powder, corosolic acid for banaba leaf extract, and 1-deoxynojirimycin for mulberry leaf extract.

### 2.4. Outcome Measures

Overnight fasting venous blood samples were collected at each visit to measure biochemical efficacy indices. Plasma HbA1c, glucose, and insulin were analyzed at the central laboratory of Seoul St. Mary's Hospital. The 75 g oral glucose tolerance test with venous blood collected at 0, 30, and 120 min was also performed. The homeostasis model assessment insulin (HOMA-IR) and the insulin sensitivity index estimated from oral glucose tolerance test (ISI_OGTT_) were calculated. Homeostasis model assessment of insulin resistance was calculated according to the formula: fasting insulin (*μ*U/mL) × fasting glucose (mmol/L)/22.5 [15]. Low-grade systemic inflammatory markers measured included intracellular adhesion molecule-1(ICAM-1), vascular cell adhesion molecule-1(VCAM-1), oxidized low-density lipoprotein (ox-LDL), plasminogen activator inhibitor-1(PAI-1), lipoprotein(Lp)(a), and high-sensitivity C-reactive protein(hs-CRP). Blood lipid profiles were also determined. The enzyme-linked immunosorbent assay (ELISA) was used to determine plasma ICAM-1 (R&D, MN, USA), VCAM-1 (R&D, MN, USA), ox-LDL (Mercodia AB, Stockholm, Sweden), LP(a) (Progen, Heidelberg, Germany), PAI-1 (R&D, MN, USA), and hs-CRP (Helica, Fullerton, CA USA). The intra- and interassay variability for inflammatory markers were 4.6 and 5.5% for ICAM-1, 3.1 and 7.0% for VCAM-1, 6.3 and 4.7% for ox-LDL, 5.0 and 8.0% for LP(a), 6.7 and 7.3% for PAI-1, and 3.8 and 4.8% for hs-CRP. Adverse effects were monitored at each visit, and serum creatinine concentration, the activity of aspartate amino transferase (AST) and alanine amino transferase (ALT) were measured as safety biochemical measures.

### 2.5. Statistical Analyses

Per-protocol analyses were conducted. Daily nutrient intake was calculated as the mean daily intake from 3-day dietary record using the Computer-Aided Nutritional Analysis Program version 3 (Korea Nutrition Society, Seoul, Korea). Statistical analyses were conducted using the SAS program version 9.1 (SAS Institute Inc., Cary, NC, USA). Descriptive data was expressed as means ± SD. For comparison of data between the two groups, we used the unpaired *t*-test for data with normal distribution and the Wilcoxon Rank Sum test for data with skewed distribution.

## 3. Result

One hundred eighteen individuals were assessed for their eligibility, and 94 individuals met the inclusion criteria. Among these participants, 32 dropped out of the study due to shortfalls in required (80%) capsule intake, changes in medication, refusal to continue, and a case of adverse event. One person on treatment dropped out of the study due to mild symptoms of muscle aching, nausea, and dry lips. The demographic and physical characteristics of study participants are shown in Table 1, and no significant difference was found due to herbal supplementation. Smoking habits, alcohol consumption, and physical activity of treatment and placebo groups were not different at baseline. No significant change was observed with daily nutrient intake from baseline to 6 months (Table 2). Body weight, BMI, waist circumference, and blood pressure measures were not significantly changed from baseline to 6 months (Table 2).

### 3.1. Blood Glucose Homeostasis Indices

Within- and between-treatment differences were assessed for herb preparation and placebo in fasting and 75 g OGTT indices of glucose and insulin (Table 3). Fasting blood glucose and insulin concentrations did not show significant differences either within- or between-treatment group. There were significant within-treatment changes in 75 g OGTT AUCg for both groups while no significance was found in between-treatment changes. There was a trend of decreased total area under the curve of insulin during the 75 g oral glucose tolerance test (75 g OGTT AUC) in treatment group; however no statistical significance was observed. Neither the within- nor the between-treatment differences in HbA1C was significant.

### 3.2. Blood Inflammatory Indices Related to Vascular Complications

Within- and between-treatment differences were assessed for herb preparation and placebo in inflammatory indices (Table 4). The plasma ICAM-1 concentration was decreased by 4.7% in treatment group and increased by 8.2% in placebo group at week 24 compared to the baseline value showing a significant between-treatment changes (*P* = 0.037). The plasma VCAM-1 and ox-LDL concentrations were decreased by 7.9% and 13.6% in treatment group at the end of study compared to those at the baseline (*P* = 0.040 and *P* = 0.003, resp.). There were significant differences in VCAM-1 and ICAM-1 concentration change between week 12 and week 24 (*P* = 0.014 and *P* = 0.048, respectively). Ox-LDL concentration was decreased at wk 24 compared to the baseline value in the treatment group (*P* = 0.003); however, no significant between group difference was found.

### 3.3. Safety Measures

There was one case of drop-out due to mild adverse effects including gastrointestinal discomfort, rashes, muscle ache, and dry lips. No significant difference was found for biochemical safety indices (data not shown).

## 4. Discussion

The present study is one of few well-controlled long-term human intervention trials to evaluate the efficacy of traditional herbs with a long historical use for diabetes. We have previously shown that the oral supplementation of these herbs effectively delays the development of insulin resistance and subsequent hyperglycemia in db/db mice [11]. Six months of supplementation with mulberry extract, Korean red ginseng root, and banaba extract (6 g/d) decreases biomarkers of systemic low-grade inflammation in subjects with impaired glucose tolerance or mild cases of type 2 diabetes. However, there was no significant improvement in blood glucose homeostasis biomarkers. Safety measures including hepatic, renal and hemostatic functions and blood pressure did not differ between the herb and placebo group. 

Excess body fat leads to insulin resistance and low grade chronic inflammatory status posing high risk of developing CVD as well as type 2 diabetes. Therefore, improvement in insulin resistance has been suggested as effective mean to delay or prevent the progress of life-style-related T2D and vascular complications. We studied the efficacy of the selected herbal preparation in subjects with impaired glucose tolerance or mild T2D because these herbs have been shown to improve insulin sensitivity and lipid metabolism which are characteristic early-phase metabolic disturbances during the development of T2D. Study results indicated that the supplementation of herbal preparations improved markers of inflammation which is related to the vascular thrombosis. There was a significant treatment difference in circulating concentrations of ICAM-1. The within-group improvements in VCAM-1 and ox-LDL concentrations were also found. 

Insulin resistance was associated with subclinical inflammation in the population who were at risk of developing T2D [16, 17]. Low-grade inflammation is a well-established risk factor of atherosclerosis [18], and this was used to explain microvascular complications frequently occurring in T2D. The oral lipid overload increased CRP and HOMA-IR in IGT subjects [19] suggesting there is an increase in the risk of vascular thrombosis before the diagnosis of diabetes. Evidences indicates that clinical cardiovascular disease can also precede development of T2D [20]. Plasma concentrations of E-selectin, ICAM-1, and VCAM-1 as markers of endothelial dysfunction were proven to predict development of T2D in initially nondiabetic women [21], and the association was independent of other known risk factors such as subclinical inflammation and obesity. Our study results also suggested that the circulating concentration of ox-LDL is improved in the treatment group. The increased oxidative stress increases the oxidized products including ox-LDL which is often elevated in T2D [22]. The overproduction of reactive oxygen species in the adipose tissue and liver of the experimental animals preceded the onset of obesity and insulin resistance [23]. ox-LDL is shown to impair adipocyte response to insulin and degrade insulin receptor substrate-1 (IRS-1) [24]. Ox-LDL is also reported to enhance atherogenecity and reduce HDL-cholesterol metabolism in T2D by suppressing lecithin-cholesterol acyltransferase activity [25]. 

We have previously shown that mulberry extract, Korean red ginseng, and banaba extract act as PPAR-*α* and PPAR-*γ* agonists improving lipid metabolism in leptin-deficient db/db mice [11]. PPARs are ligand-activated transcription factors belonging to the nuclear receptor superfamily. PPAR-*α* agonists stimulate the cellular uptake of free fatty acids, the cellular lipid storage, and the transportation of free fatty acids by regulating the expression of genes involved in lipid metabolism [26]. A previous study showed that PPAR-*γ* agonists suppressed TNF-*α*-induced ICAM-1 expression in endothelial cells [27] and vascular smooth muscle cells [28] indicating these herbs possibly reduced ICAM and VCAM concentrations possibly by acting as PPARs agonists. Polyphenols present in these herbs may have exerted antioxidant activities possibly contributing to the reduced level of ox-LDL which decreases the risk of developing insulin resistance. 

In this study, little improvement was observed in blood glucose homeostasis markers upon 6 months of supplementation with herbal preparation (6 g/d). The proportion of subjects with IGT and diabetes in this study were 56% : 44% in the treatment group and 57% : 43% in the placebo group, respectively. No significant improvement in the treatment group was found in glucose homeostasis biomarkers in the subset analyses where IGT subjects and T2D subjects were separately analyzed for the difference between the treatment and the placebo (data not shown). The large standard deviations in glucose homeostasis blood markers may be derived from the heterogeneity of study subjects. The IGT subjects were selected based on their fasting plasma glucose, and a previous report has suggested that fasting plasma glucose may not be the best predictor of insulin sensitivity [29] which is an event of metabolic dysfunction possibly regulated by the selected herbal preparation. Therefore, as indicated in study results, the fasting insulin concentration showed large variations at baseline. Low- or high-GI diet in also failed to regulate blood lipids or fasting insulin in IGT subjects [30] suggesting the efficacy evaluation of insulin-sensitizing compounds or herbal preparations may require study subjects exhibiting abnormalities more in insulin-associated metabolic features. 

In a previous clinical study to evaluate the efficacy of Korean red ginseng root preparation in type 2 diabetes patients, 6 g/d of Korean red ginseng improved OGTT-PI after 12 weeks of supplementation without concomitant improvement in glucose control [10]. This study used a combination of three different herbs which may have less ability to regulate insulin resistance compared to red ginseng itself in this study subjects. We have reported that the combination of these three herbs suppresses the development of insulin resistance in leptin-deficient db/db mouse which is an animal model of obesity-induced T2D [11]. The mode of hypoglycemic action is explained as the upregulation of liver PPAR-*α*, adipose tissue PPAR-*γ*, and lipoprotein lipase (LPL) gene expression which possibly improved lipid metabolism. The average body mass indices of our study subjects were 25.3 and 25.4 for the treatment and placebo group, and therefore, the metabolic dysregulation may have not been extensive enough to respond to the test material. In fact subjects participating in the study maintained circulating glucose and insulin concentrations that are close to normal range.

Another possible explanation for the absence of glucose control efficacy is the herb-herb interaction. Although we found no report on interactions between herbs used in this study, it has been reported that multiple interaction mechanisms exist between ingested herbs and drugs affecting their indented beneficial effects. A recent review indicated that the active constituents may compete for absorption and transportation by using same transport proteins, and herbal components can change the gastrointestinal motility and/or pH. In many cases, drug-metabolizing enzymes are targets of competition for herbal constituents and medical drugs [31].

It is important to be aware of possible drug-herb interactions because it will eventually affect efficacy and toxicity of drugs. Ginseng is known to alter anticoagulant effect of warfarin [32]. It is also reported that the concomitant use of monoamine oxidase inhibitor phenelzine and ginseng may result in insomnia, headache, tremulousness, and mania [33]. Morin, the active constituent in mulberry is shown to interact with cyclosporine, an immunosuppressant [34]. Banaba extract has been shown to increase the bioavailability of dopamine by inhibiting cytosolic sulfotrasferase [35].

There are several limitations in this study. We observed the placebo effect in insulin response markers, and this may contribute to null conclusions. Although we confirmed there was no difference in the average weight or dietary intake during the study period for both treatment and placebo arms, participants may have maintained very healthy life-styles during the study. Also, it is now well accepted that there are large individual variations in the metabolism of active compounds in herbs which may have caused large intraindividual variations to exert biological activities. A recent review indicated that agricultural pollutants such as organophosphates and organochlorines affect cellular metabolism of carbohydrate and lipid thereby developing insulin resistance and impaired glucose homeostasis [36]. Alterations of enzyme activities and the increased oxidative stress have been suggested as possible mechanisms of action. Although the test herbs used in this study met the food-grade quality, appropriate studies to elucidate possible interactions between pollutants and glucose/lipid metabolism are needed. Due to a long supplementation period, we adopted a parallel design instead of the cross-over design which may have created large interindividual variations. Finally this study does not provide mechanistic explanations or information on active compounds, which need further investigation.

## 5. Conclusion

A long-term supplementation of mulberry, Korean red ginseng, and banaba leaf improved low-grade systemic inflammation without significant efficacy in blood glucose homeostasis control. The fast growing incidence of T2D requires early detection and prevention. The complementary use of dietary supplement may be used to delay the disease progression at early stage. The chemical complexity of herbal products and large interindividual responses needs to be considered carefully in future clinical trials.

## Figures and Tables

**Table 1:** Basic characteristics of study subjects.

	Herb group (*n* = 32) (%)	Placebo group (*n* = 30) (%)
Age (year)	58.9 ± 8.9^(1)^	56.6 ± 9.6
Sex (Man %)	32 (68.75)^(2)^	30 (73.33)
Height (cm)	164.3 ± 7.9	165.8 ± 7.9
Weight (kg)	68.5 ± 12.1	69.9 ± 9.8
BMI (kg/m^2^)^(3)^	25.3 ± 3.4	25.4 ± 2.5
Waist (cm)	89.1 ± 6.8	91.2 ± 6.3
Blood pressure (mHg)		
Systolic	118.9 ± 15.1	129.7 ± 13.5
Diastolic	80.2 ± 10.4	82.8 ± 7.0
Smoking status		
Yes	8 (25)	6 (20)
No	24 (75)	24 (80)
Alcohol consumption		
Yes	17 (53.12)	20 (66.67)
No	15 (46.88)	10 (33.33)
Frequency of exercise		
None	4 (12.50)	5 (16.66)
1-2/week	5 (15.63)	8 (26.66)
3-4/week	10 (31.25)	5 (16.67)
5-6/week	7 (21.88)	1 (3.33)
Almost everyday	6 (18.75)	11 (36.67)

^(1)^Mean ±SD.

^(2)^Number of subjects (%).

^(3)^Body mass index.

**Table 2:** Follow-up changes in anthropometry and nutrient intake.

		Herb group (*n* = 32)	Placebo group (*n* = 30)
Weight (kg)	Baseline	68.5 ± 12.1^(1)^	69.9 ± 9.8
	Week 24	67.6 ± 12.7	68.3 ± 9.4

BMI (kg/m^2^)^(2)^	Baseline	25.3 ± 3.4	25.4 ± 2.5
	Week 24	24.9 ± 3.6	24.8 ± 2.4

Waist (cm)	Baseline	89.1 ± 6.8	91.2 ± 6.3
	Week 24	87.8 ± 7.3	88.8 ± 6.9

Blood pressure (mmHg)	Baseline (sys.)	118.9 ± 15.1	129.7 ± 13.5
	Week 24 (sys.)	113.1 ± 12.0	119.2 ± 13.4
	Baseline (dia.)	80.2 ± 10.4	82.8 ± 7.0
	Week 24 (dia.)	75.9 ± 8.7	75.8 ± 8.1

Energy (kcal)	Baseline	1710.1 ± 375.6	1657.9 ± 396.1
	Week 24	1700.5 ± 336.3	1734.5 ± 427.6

Animal protein (g)	Baseline	34.8 ± 18.0	33.7 ± 18.9
	Week 24	36.9 ± 23.3	39.2 ± 26.3

Plant protein (g)	Baseline	37.7 ± 10.9	34.8 ± 8.7
	Week 24	38.5 ± 11.7	34.5 ± 9.6

Carbohydrate (g)	Baseline	257.2 ± 60.6	232.5 ± 54.0
	Week 24	245.5 ± 49.5	242.8 ± 58.8

Animal fat (g)	Baseline	21.4 ± 15.8	23.4 ± 19.0
	Week 24	26.5 ± 22.8	25.8 ± 19.5

Plant fat (g)	Baseline	20.3 ± 11.9	17.7 ± 9.2
	Week 24	20.2 ± 11.2	19.1 ± 9.5

^(1)^Mean ± SD.

^(2)^Body mass index.

**Table 3:** Between treatment differences in blood glucose homeostasis indices following 6-month supplementation with herbal preparation or placebo in subjects with IGT or mild T2D.

	Treatment (*n* = 32)			Placebo (*n* = 30)		
	Baseline	12 week	24 week	Baseline	12 week	24 week
Fasting glucose^1^	6.93 ± 1.15	7.04 ± 1.19	6.79 ± 1.37	7.54 ± 1.47	7.4 ± 1.49	7.04 ± 1.08
Fasting insulin^2^	9.65 ± 5.45	11.03 ± 8.70	9.67 ± 4.57	8.93 ± 4.98	9.43 ± 5.05	9.32 ± 6.15
HOMA-IR	2.99 ± 1.89	3.61 ± 3.44	2.92 ± 1.54	3.02 ± 1.72	3.15 ± 1.80	2.83 ± 1.73
OGTT AUC_g_^3^	23.83 ± 5.51	23.26 ± 5.15	21.87 ± 5.08^†^	25.27 ± 6.12	24.64 ± 4.79	23.27 ± 4.65^†^
OGTT AUC_i_^4^	93.79 ± 62.77	88.67 ± 55.55	81.55 ± 44.32	81.1 ± 50.71	74.75 ± 53.28	88.79 ± 76.88
ISI_OGTT_	5.97 ± 6.13	5.72 ± 4.69	5.57 ± 3.66	5.51 ± 4.68	5.69 ± 4.40	6.44 ± 6.34
HbA_1c_ (%)	6.37 ± 0.60	6.39 ± 0.71	6.45 ± 0.67	6.52 ± 0.74	6.52 ± 0.86	6.54 ± 0.70

		Mean difference (treatment versus placebo)				
		**0–12**	** 12–24**	**0–24**		

Fasting glucose^1^		−0.24 ± 1.56	−0.11 ± 1.52	−0.35 ± 1.54		
Fasting insulin^2^		−0.89 ± 8.86	1.26 ± 10.45	0.37 ± 7.08		
HOMA-IR		−0.49 ± 3.21	0.37 ± 3.59	−0.12 ± 2.07		
OGTT AUC_g_^3^		−0.06 ± 6.52	0.03 ± 5.50	−0.03 ± 5.71		
OGTT AUC_i_^4^		−1.24 ± 56.74	21.16 ± 53.85	19.92 ± 66.28		
ISI_OGTT_		0.43 ± 6.85	0.89 ± 6.12	1.32 ± 8.58		
HbA_1c_ (%)		−0.03 ± 0.68	−0.03 ± 0.81	−0.06 ± 0.76		

^1^ *u*M/mL, ^2^ *μ*U/mL, ^3^ *u*M/mL, ^4^ *μ*U/mL.

**^†^**significantly different within-treatment change from baseline (*P* < 0.05, paired *t*-test).

**Table 4:** Between treatment differences in inflammation indices following 6-month supplementation with herbal preparation or placebo in subjects with IGT or mild T2D.

	Treatment (*n* = 32)			Placebo (*n* = 30)		
	Baseline	12 week	24 week	Baseline	12 week	24 week
ICAM (ng/mL)	224.4 ± 76.82	197.0 ± 67.5^∗*¶*^	213.8 ± 85.19	218. 7 ± 102.6	199.0 ± 85.10^†^	236.7 ± 109.6
VCAM (ng/mL)	668.4 ± 171.5	651.8 ± 174.3	615.3 ± 201.8^†^	701.2 ± 223.9	660.9 ± 228.0	707.9 ± 315.1
ox-LDL (U/I)	60.2 ± 17.55	63.82 ± 18.28	51.98 ± 13.99^‡^	60.82 ± 20.49	60.77 ± 20.81	54.68 ± 13.62
Lp(a) (mg/dL)	25.34 ± 16.25	22.91 ± 17.80	26.98 ± 19.77	26.96 ± 19.41	24.01 ± 19.04^†^	25.65 ± 19.95
PAI-1 (ng/mL)	5.65 ± 3.33	6.61 ± 4.69	5.59 ± 3.84	7.55 ± 12.36	6.21 ± 4.56	6.37 ± 5.27
Hs-CRP (ng/mL)	1.64 ± 1.90	1.66 ± 1.18	1.33 ± 1.42	2.05 ± 2.94	1.55 ± 1.74	1.87 ± 2.96

		Mean difference (treatment versus placebo)				
		**0–12**	**12–24**	**0–24**		

ICAM (ng/mL)		7.73 ± 57.54	20.89 ± 59.7^**a**^	28.62 ± 72.94^**b**^		
VCAM (ng/mL)		−23.6 ± 184.1	83.39 ± 199.2^**a**^	59.76 ± 244.9		
ox-LDL (U/I)		−3.67 ± 21.78	5.74 ± 18.71	2.07 ± 17.65		
Lp(a) (mg/dL)		−0.52 ± 10.19	−2.43 ± 13.34	−2.95 ± 12.87		
PAI-1 (ng/mL)		−2.3 ± 13.00	1.18 ± 5.65	−1.12 ± 13.13		
Hs-CRP (ng/mL)		−0.52 ± 3.97	0.65 ± 2.54	0.13 ± 4.25		

*significantly different within-treatment change from baseline (*P* < 0.05, paired *t*-test) ^†^< 0.05 ^‡^< 0.01 ^¶^< 0.001.

^a^significantly different between-treatment change during week 12–24 (*P* < 0.05, the Wilcoxon rank sum test).

^b^significantly different between-treatment change during week 0–24 (*P* < 0.05, the Wilcoxon rank sum test).
